# *Molecules’* Highlights in 2008 and a Look Forward to 2009

**DOI:** 10.3390/molecules14010584

**Published:** 2009-01-23

**Authors:** Shu-Kun Lin, Derek McPhee

**Affiliations:** Molecular Diversity Preservation International (MDPI), Kandererstrasse 25, Basel CH-4057 Switzerland; Tel. +41 61 683 7734, Fax: +41 61 302 8918, E-mails: lin@mdpi.org; mcphee@mdpi.org; http://www.mdpi.org/lin

As has now become a custom for *Molecules*, as one year ends and another starts its Publisher and Editor-in-Chief would like to take stock of our progress in 2008 and offer our readership a preview of things to come in 2009. Just as we had hoped in last year’s editorial [1], the year that just concluded proved to be another outstanding one for MDPI's flagship journal *Molecules*. By year’s end we had published 239 papers and three Editorials or Editor's Notes for a total of 3,252 pages. While the 8% growth in the number of papers published compared to 2007 may appear modest, the number of pages published represented a 22% increase over the prior year, and was largely attributable to the increase in the number of reviews published. 

Last year also saw the passing of the submission deadlines for some of the special issues carried over from the previous year, like those dedicated to *Phenolics and Polyphenolics*, *5-Fluorouracil*, *Prodrugs*, *Alkaloids* and *Spirocompounds*, whose Guest Editors, Professors Pereira and Andrade, Puoci, Vanden Eynde, Valentao and Plourde shall continue to work on the manuscripts submitted for these issues that will continue to appear in the coming months, as they make their way through the peer review process. These Guest Editors will be joined in 2009 by those from our lengthy list of planned new special issues: *Macromolecules Applied to Pharmaceutics* (Coviello); *Dynamic Combinatorial Chemistry* (Otto); *Acetogenins: Extraction, Synthesis and Biological Properties* (Figadere); *Coumarins and Xanthones* (Richomme); *Neglected Diseases: Medicinal Chemistry and Natural Product Chemistry* (Batista); *Nucleic Acids* (Carlsen); *Triterpenes and Triterpenoids* (Roussis); *Biodegradation: Organic, Medicinal and Analytical Chemistry* (Lin and Teramoto); *Dendrimers – From Synthesis to Applications* (Zhang); *Ferrocenes* (Ríos Torres); *Microwave Assisted Synthesis* (Seijas Vázquez); *Organocatalysis* (Zhong); *Quinones and Hydroquinones: Target Molecules and Building Blocks* (Spyroudis); *Selenium and Tellurium Chemistry* (Pietschnig); *Alpha- or Beta-aminophosphonates and -phosphinate*s (Palacios); *Aporphines and Oxaporphines* (Sobarzo-Sánchez); *Baylis-Hillman Reaction and Related Processes* (Li); *Ionic Liquids* (Bonrath) and *Sonochemistry – Organic Synthesis* (Bonrath). 

We are also happy to note that as the Publisher anticipated in a previous Editorial [2], the move to a full Open Access format was indeed reflected positively in our 2007 Impact Factor, which was 0.940 (up from 0.841 in 2006) and based on the data currently available from SCI, we expect that the 2008 IF will be around 1.2 or slightly higher. In addition *Molecules* is now indexed and/or abstracted in PubMed/Medline; Beilstein CrossFire; Chemical Abstracts (CAplus); Science Citation Index Expanded; Chemistry Citation Index; Current Contents/Physical, Chemical & Earth Sciences; SciSearch; Research Alert; Scopus; Google Scholar; IndexCopernicus; Journal Citation Report; Scirus; Directory of Open Access Journals (DOAJ) and Open-J-Gate, so authors may be assured of the prompt and widest possible dissemination of their results. 

Also in 2008 we renewed the composition of our General Editorial Board, which had remained essentially unchanged for the past 13 years since we started publication. We are extremely pleased to report that new names of the highest caliber have been added, while many of the luminaries already on our Board, including three Nobel Prize winners, graciously agreed to extend their relationship with *Molecules*. 

Finally, readers will have noticed our move to a new publication platform at www.mdpi.com, where we offer an appealing new layout complete with graphical abstracts, powerful search and document export features and access to papers within 1-2 days of acceptance for publication, rather than on a monthly basis when the issues are released as was previously the case. In 2008, DOI numbers were added for all papers published in MDPI journals. Though all the pdf files of the papers published and indexed in many databases are still available from the www.mdpi.org server, complete sets of the all the papers are now available at www.mdpi.com. This progress can be duly attributed to Dietrich Rordorf and his software development and data management team members Marc Bigler, Robert Seibert and Daniel Schmutz.

In conclusion, as 2009 begins, we take this opportunity to offer our best wishes and thanks to our many authors, anonymous peer-reviewers and readers for their continued support of our efforts and our Assistant Editors Ms Felicity Wright and Ms Jely He at our Basel and Beijing offices, respectively, for their hard work handling the submission and peer review process. As always, we welcome your comments on these matters. Messages with a suitable subject header may be sent to lin@mdpi.org.
